# Prognostic value of PET/CT and MR-based baseline radiomics among patients with non-metastatic nasopharyngeal carcinoma

**DOI:** 10.3389/fonc.2022.952763

**Published:** 2022-10-24

**Authors:** Roshini Kulanthaivelu, Andres Kohan, Ricarda Hinzpeter, Zhihui Amy Liu, Andrew Hope, Shao Hui Huang, John Waldron, Brian O’Sullivan, Claudia Ortega, Ur Metser, Patrick Veit-Haibach

**Affiliations:** ^1^ Joint Department of Medical Imaging, University Health Network, Mount Sinai Hospital and Women’s College Hospital, University of Toronto, Toronto, ON, Canada; ^2^ Department of Biostatistics, Princess Margaret Cancer Centre, University Health Network, Dalla Lana School of Public Health, University of Toronto, Toronto, ON, Canada; ^3^ Department of Radiation Oncology, University Health Network, Mount Sinai Hospital and Women’s College Hospital, University of Toronto, Toronto, ON, Canada

**Keywords:** NPC, Radiomics, PET/CT, MRI, prognosis

## Abstract

**Purpose:**

Radiomics is an emerging imaging assessment technique that has shown promise in predicting survival among nasopharyngeal carcinoma (NPC) patients. Studies so far have focused on PET or MR-based radiomics independently. The aim of our study was to evaluate the prognostic value of clinical and radiomic parameters derived from both PET/CT and MR.

**Methods:**

Retrospective evaluation of 124 NPC patients with PET/CT and radiotherapy planning MR (RP-MR). Primary tumors were segmented using dedicated software (LIFEx version 6.1) from PET, CT, contrast-enhanced T1-weighted (T1-w), and T2-weighted (T2-w) MR sequences with 376 radiomic features extracted. Summary statistics describe patient, disease, and treatment characteristics. The Kaplan–Meier (KM) method estimates overall survival (OS) and progression-free survival (PFS). Clinical factors selected based on univariable analysis and the multivariable Cox model were subsequently constructed with radiomic features added.

**Results:**

The final models comparing clinical, clinical + RP-MR, clinical + PET/CT and clinical + RP-MR + PET/CT for OS and PFS demonstrated that combined radiomic signatures were significantly associated with improved survival prognostication (AUC 0.62 *vs* 0.81 *vs* 0.75 *vs* 0.86 at 21 months for PFS and 0.56 *vs* 0.85 *vs* 0.79 *vs* 0.96 at 24 months for OS). Clinical + RP-MR features initially outperform clinical + PET/CT for both OS and PFS (<18 months), and later in the clinical course for PFS (>42 months).

**Conclusion:**

Our study demonstrated that PET/CT-based radiomic features may improve survival prognostication among NPC patients when combined with baseline clinical and MR-based radiomic features.

## Introduction/Background

Nasopharyngeal carcinoma (NPC) is an epithelial malignancy arising from the mucosa of the nasopharynx, and it accounts for 0.7% of all malignancies (1). NPC affects less than one person per 100,000 in North America (2), but is endemic in Southern China, the Middle East, and North Africa (2). Although the prognosis of NPC is largely good, with 5-year survival rates reaching up to 80% (3), 20%–30% of patients experience treatment failure from locoregional recurrence or distant metastasis (4).

Radiotherapy with or without concurrent chemotherapy is regarded as the standard of care for NPC, and accurate staging, including optimized imaging, is crucial for appropriate treatment stratification (5). MR assessment is performed due to superior soft tissue contrast resolution compared with CT, and ^18^Fluoride-Fluorodeoxyglucose-Position Emission Tomography/Computed Tomography (PET/CT) is utilized to evaluate for both the presence of a primary lesion in cases of diagnostic uncertainty, and for the presence of local lymph node and distant metastatic disease. Increasing stages have been demonstrated to be associated with poorer prognosis (3, 6). However, if these patients are identified early, escalated therapy strategies can be employed.

Outside of conventional TNM staging, there is no consensus on specific prognostic biomarkers that can potentially improve survival among NPC patients (4). Various clinical factors such as EBV titer, hemoglobin, LDH, CRP, neutrophil to lymphocyte ratio, and platelet counts have been identified as factors potentially associated with poor survival (6, 7). However, the clinical utility of these parameters, outside of EBV titer (4), is limited and new tools are required to identify patients at risk of poor prognosis. In recent years, radiomics has emerged as a promising field that can potentially provide a means of improved prognostication.

Radiomics is an extension of computer-aided diagnosis and detection and relies upon the concept that “medical images contain information about disease-specific processes that are imperceptible to the human eye” (8). Images are converted to mineable data that are analyzed using computer algorithms both quantitatively in terms of the spatial distribution of signal intensities and pixel interrelationships and qualitatively in terms of differences in intensity, shape, or texture (8–10).

Multiple studies, dating as far back as 2017, have demonstrated that multiparametric MR-based radiomic parameters can be utilized to predict prognosis, progression-free survival (PFS), and recurrence in patients with advanced NPC (6, 11–19) and non-metastatic NPC (20, 21) with superior prognostic performance over TNM staging (17, 22).

Metabolic parameters derived from PET/CT have revolutionized oncological imaging (7). In terms of radiomic analysis, more recent studies have utilized radiomic features from baseline PET/CT to quantitatively characterize intra-tumoral heterogeneity and provide prognostic information among patients with NPC, with the prediction of locoregional recurrence and distant metastasis in advanced NPC (7, 23–26).

There have not, however, been any studies in the literature so far that have evaluated the combined prognostication value between radiomic signatures on both PET/CT and MR and clinical parameters among patients with NPC. The aim of this study was to therefore evaluate and compare the prognostic value of clinical data, radiomic features extracted from PET/CT and MR both separately and combined.

## Materials and methods

This retrospective study was approved by the institutional review board and the need to obtain informed consent from patients was waived.

### Patient selection

A total of 146 patients with pathologically confirmed NPC (Stages I–IVC) underwent staging with PET/CT between December 2012 and July 2018 at the University Hospital Network, Toronto. Of these, 130 patients had undergone MR for the purpose of radiotherapy planning (RP-MR). Six patients with stage M1 (treated with palliative intent) were excluded. Subsequently, 124 patients with curative therapeutic intent with both PET/CT and RP-MR scans were included for analysis.

Demographic details (age, sex), as well as clinical variables including ECOG, smoking history, pathology, EBER, EBV titer, HPV, TNM staging, date of diagnosis and last follow up, treatment intent and regimen, RT dates, dose, and follow up data including local, regional, or distant failure, date, and status at last follow up were collated and are summarized in **Table 1**. Staging was performed according to the American Joint Committee on Cancer TNM Staging System Manual, 7th edition. Patient follow-up was measured from the date of diagnosis to the last day of follow up. Overall Survival (OS) time was defined as at the time from the date of diagnosis to the date of death or last follow-up, with PFS time defined from the date of diagnosis to the date of local, regional, or distant failure, or death/last follow-up.

**Table 1:** Population characteristics.

	n = 124
Sex (n)	
Male	84% (104)
Female	16% (20)
mean Age in years (SD)	54.8 (11.6)
Smoking History (n)	
Current	21% (26)
Ex-Smoker	26% (32)
Never	50% (62)
Unknown	3% (4)
Primary (n)^*^	
NPC Type 1/2	25% (31)
NPC Type 3	75% (93)
Viral State (n)	
EBER +	89% (110)
HPV +	6% (8)
Non-Viral	3% (4)
Unknown	2% (2)
mean EBV Titer (IU/ml, SD)	30,433.3 (175,831.1)
T Stage (n)^‡^	
1/2	43% (54)
3	31% (38)
4	26% (32)
N Stage (n)^‡^	
0	15% (18)
1	32% (40)
2	43% (53)
3	10% (13)
Overall Stage (n)^‡^	
I	7% (9)
II	13% (16)
III	46% (57)
IV	1% (1)
IVA	23% (28)
IVB	10% (13)
RT/CRT Regimen (n)	
CCRT − RT	36% (45)
CCRT + AC – IC + CCRT	64% (79)

^*^WHO classification ^‡^7th edition UICC/AJCC staging system, CCRT, concurrent chemoradiation therapy; RT, Radiation Therapy, AC, adjuvant chemotherapy; IC, induction chemotherapy.

### Image acquisition

#### PET

Pretreatment whole-body PET/CT was acquired on a Siemens mCT40 PET/CT scanner (Siemens Healthineers, Erlangen, Germany). Patients were positioned supine with images obtained from the top of the skull to the upper thighs. Iodinated oral contrast material was administered for bowel opacification; no intravenous iodinated contrast material was used. Patients were injected with 300–400 MBq (4–5 MBq/kg) of ^18^Fluoride-Fluorodeoxyglucose (^18^F-FDG) after having fasted for 6 h, and PET/CT scanning was performed after approximately 60 min. Overall, five to nine bed positions were obtained, depending on patient height, with an acquisition time of 2–3 min per bed position. The CT settings were as follows: 120 kV; 3.0 mm slice width; 2.0 mm collimation; 0.8 s rotation time; and 8.4 mm feed/rotation. A PET emission scan using time of flight with scatter correction was obtained, covering the identical transverse field of view. The PET parameters were as follows: image size: 2.6 pixels; slice: 3.27; and a 5-mm full width at half-maximum (FWHM) gaussian filter type. Overall, patient data has been acquired as published by our group previously (27).

#### RP-MRI

All patients were examined on a 3.0T MRI scanner for radiotherapy planning (Siemens Magnetom Verio syngo MR B17, Siemens Healthineers, Erlangen, Germany). Post contrast T1-weighted (T1-w) and T2-weighted (T2-w) MR images were acquired with the following parameters: axial T1-w turbo spin-echo fat saturated images post contrast (TR 1,240 ms/TE 11 ms, ET 256 × 205, FOV 24 × 24 cm, slice thickness 3 mm) and axial T2-w turbo spin-echo fat saturated images (TR 8,290 ms, TE 117 ms, ET 22, FOV 24 × 24 cm, slice thickness 3 mm).

### Radiomic feature extraction

Radiomic features were extracted using the LIFEx platform version 6.1 (IMIV/CEA, Orsay, France) (28) from axial PET, low-dose unenhanced CT (acquired as part of the PET/CT), axial fat saturated and contrast-enhanced T1-w and T2-w RP-MR Digital Imaging and Communications in Medicine (DICOM) images that had been archived in PACS (**Table 1 Supplemental Material**). Semi-automatic segmentation of the PET component was performed using a thresholding method, with minor manual correction as required. PET volumes of interest (VOI) were defined based on (a) background threshold; (b) threshold at 40%; and (c) threshold at 70% of the SUVmax. Volumetric segmentation of the tumor on CT and MRI was carried out manually. Because there is no thresholding method available for the CT or MR component, the contours for the CT-derived VOI were drawn manually in a slice-by-slice fashion to cover the entire tumor. The minimal VOI included at least 64 voxels and was confirmed (by the “CheckTex” feature in the software) to make sure it created a single contiguous piece that enabled consistent textural feature calculation.

To account for the impact of different resampling schemes in MR, a fixed bin width of 128 bins, which corresponded to absolute resampling, was chosen after the initial sampling of healthy normal tissue (masseter muscle) for reference (29). Segmentation was performed by one radiologist with 7 years of experience (RK). Only primary lesions were considered in the study; lymph nodes or secondary lesions were not included. A total of 94 radiomic features were obtained from each imaging sequence.

### Statistical analysis and modeling

Summary statistics were used to describe patient, disease, and treatment characteristics. The Kaplan–Meier (KM) method was used to estimate overall survival (OS) and progression-free survival (PFS).

Preprocessing of the radiomic data included removing features with more than 50% missing observations, i.e., due to too few voxels to analyze, and removing features with little variation (those with <4 unique values). The value of the 99.9 percentile was used to cap the upper extreme values for each feature. All features were standardized with a mean of zero and a standard deviation of one.

Clinical factors were selected based on statistical significance with a p-value <0.05 in the univariable analyses (UVA) to build multivariable Cox proportional hazards models for OS and PFS, respectively. Subsequently, each radiomic feature was added to the clinical model, and features with a p-value <0.01 were selected for correlation assessment to filter out highly correlated features using the *caret* (30) package in R. If the absolute pairwise correlation was higher than 0.5, then the feature with the larger mean absolute correlation was removed. The final model included both clinical variables and radiomic features. Model performance was quantified and visualized using the area under the time-dependent ROC curve (AUC) (31) calculated using leave-one-out cross-validation. All statistical analyses were conducted in R version 4.0.2 (32).

## Results

### Population characteristics

Out of the 124 patients analyzed, 84% (n = 104) were males, 50% (n = 62) had never smoked, and 95% (n = 118) had had previous infection by either HPV or EBV, with a mean EBV titer of 30,433.5 IU/ml. The mean age was 54.8 y ( ± 11.6) and the majority had stage III disease or lower (66%, n = 82) (**Table 1**).

**Figure 1:**
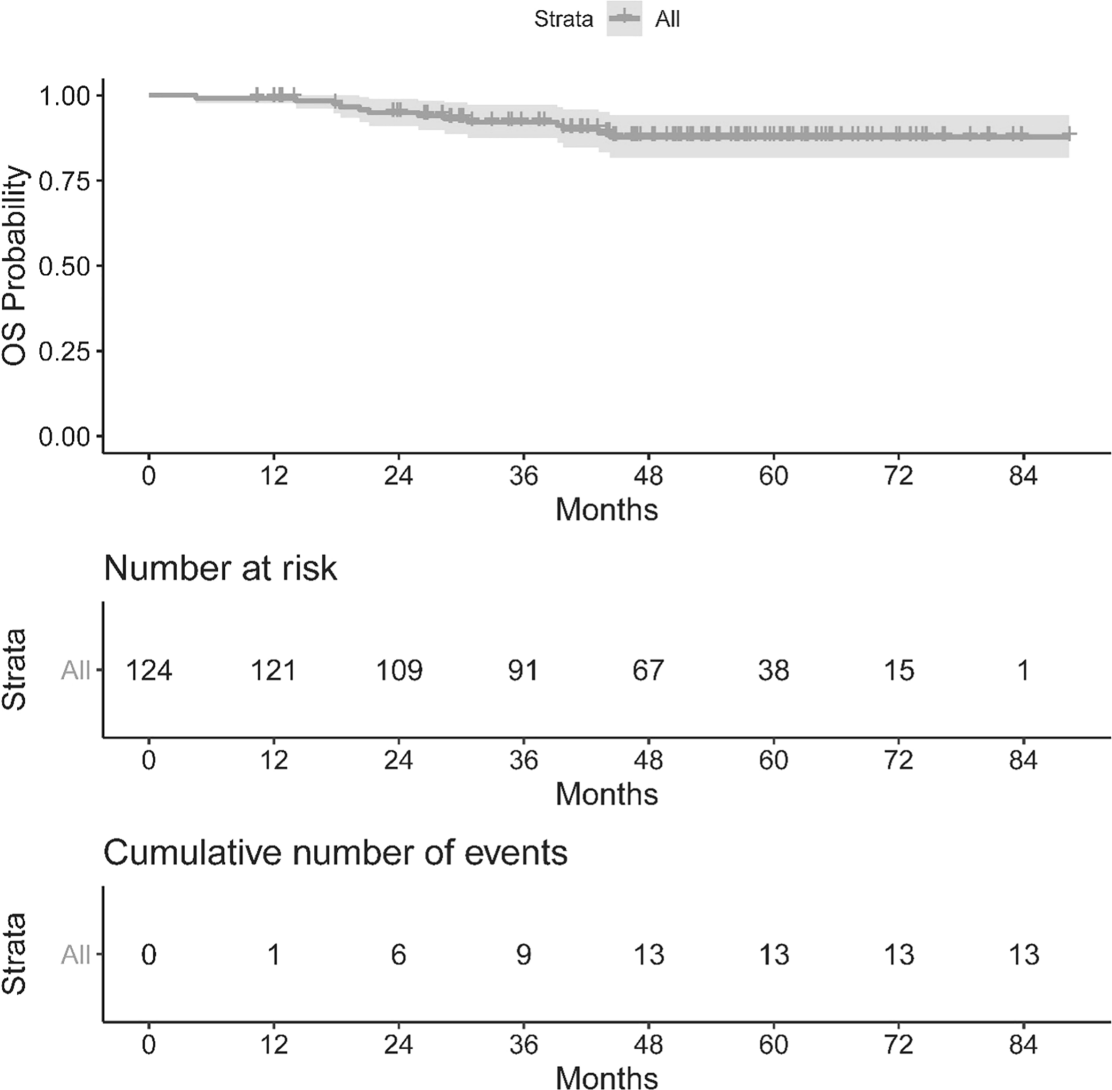
Kaplan–Meier curve for Overall Survival.

**Figure 2:**
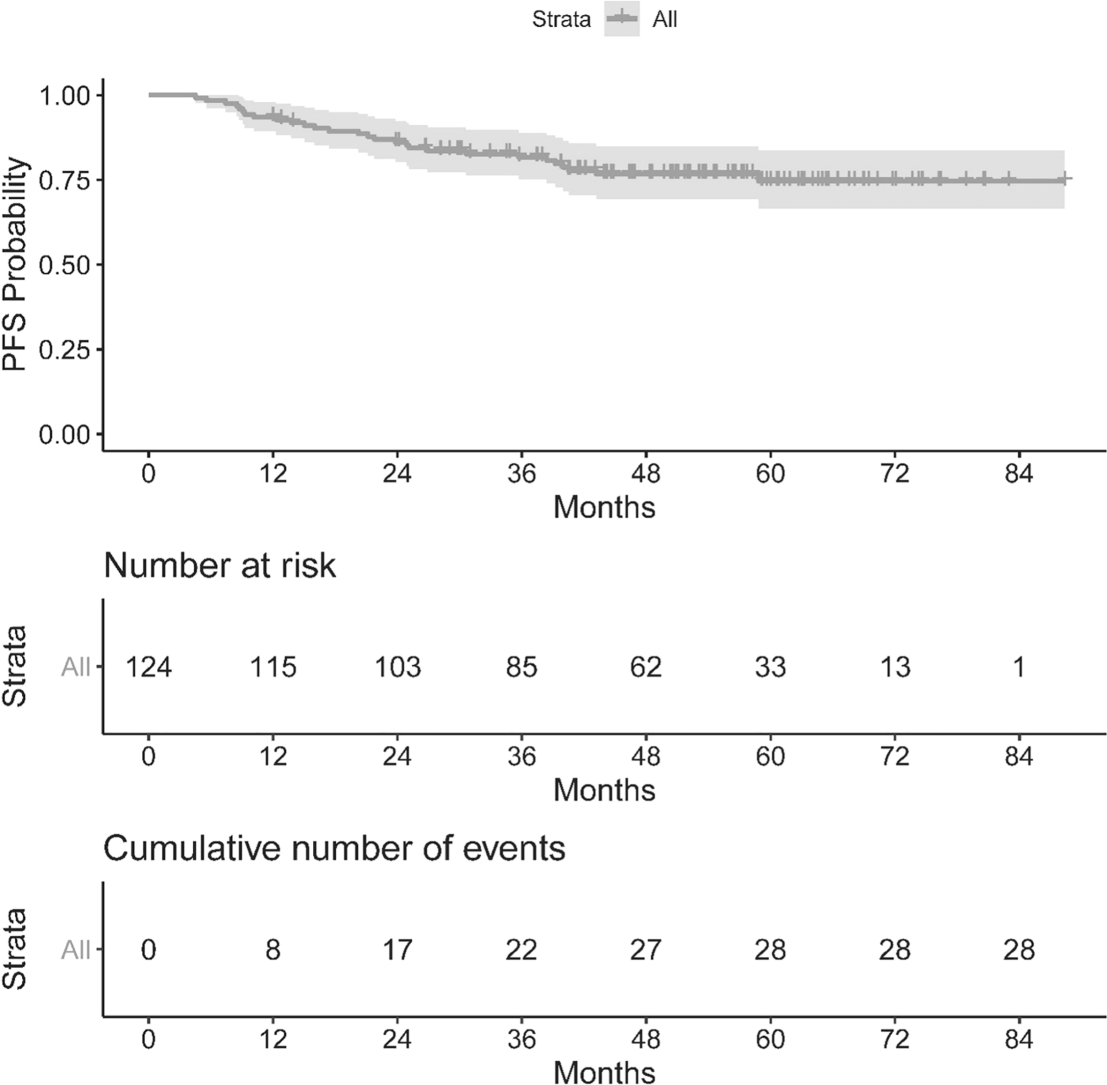
Kaplan Meier curve for Progression Free Survival.

The OS and PFS of our population can be seen in **Figures 1**, **2**. The median follow up period was 50.3 months (a range of 4.5 to 88.3 months). Overall, 13 cancer-related deaths and 28 patients with relapse of their index disease were noted during the follow-up period. In both cases, most of these events (100% for OS and 96% for PFS) happened before 48 months.

### Statistical analysis of prognostic factors

Univariable statistical analysis was performed for the clinical variables, as shown in **Table 2**. Age was found to be significant for OS, and both age and treatment regimen were found to be significant for PFS, and thus these variables were included in the final multivariable models. On top of the selected clinical variables, statistically significant radiomic features with a p-value <0.01 are shown in **Table 3** for OS and **Table 4** for PFS.

**Table 2:** Univariable analysis of clinical variables.

	OS			PFS		
Covariate	HR (95% CI)	p-value	Global p-value	HR (95%CI)	p-value	Global p-value
Age (years)	1.05 (1.00, 1.10)		0.043	1.05 (1.01, 1.08)		0.0046
Sex			1			0.19
Female	Reference			Reference		
Male	Not estimable			2.64 (0.63, 11.11)		
ECOG PS			0.074			0.33
ECOG 0	Reference			Reference		
ECOG 1-2	2.70 (0.91, 8.04)			1.46 (0.68, 3.12)		
Smoking pack year	1.01 (0.98, 1.05)		0.38	1.01 (0.99, 1.03)		0.51
History of Smoking			0.92			0.67
Current	Reference			Reference		
Ex-smoker	1.83 (0.34, 10.01)	0.48		2.05 (0.63, 6.65)	0.23	
Non-smoker	1.40 (0.29, 6.74)	0.67		1.46 (0.48, 4.45)	0.5	
Unknown	Not estimable	1		1.91 (0.21, 17.14)	0.56	
Primary Pathology			0.55			0.97
NPC, Type 1/2 (WHO I/IIA)	Reference			Reference		
NPC: Type 3 (WHO IIB)	0.70 (0.22, 2.27)			0.98 (0.42, 2.31)		
EBER			0.37			0.095
Negative	Reference			Reference		
Positive	0.50 (0.11, 2.29)			0.44 (0.17, 1.16)		
EBV Titer pre RT	1.00 (1.00, 1.00)		0.59	1.00 (1.00, 1.00)		0.59
T stage 7th			0.46			0.43
T1–2	Reference			Reference		
T3	1.39 (0.35, 5.56)	0.64		0.63 (0.24, 1.66)	0.35	
T4	2.28 (0.61, 8.48)	0.22		1.24 (0.53, 2.91)	0.62	
N stage 7th			0.75			0.93
N0	Reference			Reference		
N1	0.43 (0.09, 2.11)	0.3		1.52 (0.42, 5.54)	0.52	
N2	0.69 (0.17, 2.78)	0.61		1.44 (0.41, 5.12)	0.57	
N3	0.46 (0.05, 4.45)	0.5		1.59 (0.32, 7.87)	0.57	
Overall Stage 7th			0.27			0.18
I-III	Reference			Reference		
IV	1.85 (0.62, 5.50)			1.67 (0.79, 3.54)		
RT/CRT Regimen			0.32			0.035
CCRT - RT	Reference			Reference		
CCRT+AC - IC+CCRT	0.58 (0.19, 1.72)			0.45 (0.21, 0.95)		

**Table 3:** Feature selection for OS.

Covariate	HR	95% CI lower BOUND	95% CI upper BOUND	p-value
PET_CONVENTIONAL_SUVbwQ1*	1.81	1.15	2.84	0.00981
PET_CONVENTIONAL_SUVbwQ2	1.83	1.17	2.86	0.00808
PET_CONVENTIONAL_TLG.mL.onlyForPETorNM.	1.72	1.15	2.59	0.00862
PET_DISCRETIZED_SUVbwQ1	1.88	1.20	2.96	0.00627
PET_DISCRETIZED_SUVbwQ2	1.82	1.17	2.85	0.00825
PET_DISCRETIZED_TLG.mL.onlyForPETorNM.	1.75	1.16	2.63	0.00781
PET40_CONVENTIONAL_TLG.mL.onlyForPETorNM.	1.77	1.18	2.64	0.00573
PET40_DISCRETIZED_TLG.mL.onlyForPETorNM.	1.80	1.20	2.71	0.00449
PET40_GLZLM_GLNU	1.76	1.18	2.62	0.00572
CT_GLZLM_ZLNU	1.69	1.13	2.53	0.00991
RP_T1_SHAPE_Volume.vx.	1.67	1.28	2.19	0.00019
RP_T1_GLRLM_LRE	1.57	1.14	2.18	0.00634
RP_T1_GLRLM_GLNU	1.83	1.38	2.43	0.00002
RP_T1_NGLDM_Busyness	1.60	1.18	2.17	0.00234
RP_T1_GLZLM_GLNU*	1.68	1.15	2.46	0.00688

*Chosen variables for the model after correlation analysis.

**Table 4:** Feature selection for PFS.

Covariate	HR	95% CI lower BOUND	95% CI upper BOUND	p-value
PET_CONVENTIONAL_SUVbwmin	1.78	1.34	2.37	0.00008
PET_CONVENTIONAL_SUVbwQ1	1.90	1.38	2.61	0.00007
PET_CONVENTIONAL_SUVbwQ2	1.71	1.23	2.38	0.00157
PET_DISCRETIZED_SUVbwmin*	1.80	1.35	2.40	0.00006
PET_DISCRETIZED_SUVbwQ1	1.94	1.40	2.67	0.00006
PET_DISCRETIZED_SUVbwQ2	1.72	1.23	2.39	0.00133
PET_GLZLM_SZLGE	0.49	0.29	0.84	0.00884
RP_T1_CONVENTIONAL_Skewness*	1.64	1.16	2.31	0.00538
RP_T1_GLRLM_GLNU	1.49	1.14	1.94	0.00393
RP_T1_NGLDM_Busyness*	1.41	1.10	1.82	0.00766

*Chosen variables for the model after correlation analysis.

After filtering out highly correlated features, the final models are presented in **Table 5**. For OS, age (p = 0.026), PET_CONVENTIONAL_SUVbwQ1 (p = 0.009), and RP_T1_GLZLM_GLNU (p = 0.006) were significant prognostic factors, while for PFS PET DISCRETIZED SUVbwmin (0.006) and RP T1 NGLDM Busyness (p = 0.043) were significant prognostic factors.

**Table 5:** Final prognostic models for PFS and OS.

Final Model for OS			RT MRI Model for PFS		
Covariate	HR (95% CI)	p-value	Covariate	HR (95%CI)	p-value
Age	1.06 (1.01, 1.11)	0.026	Age	1.04 (1.00, 1.08)	0.06
PET CONVENTIONAL SUVbwQ1	1.92 (1.18, 3.13)	0.0092	Regimen = CCRT + AC − IC+CCRT (vs CCRT − RT)	0.63 (0.27, 1.47)	0.28
RMP T1 GLZLM GLNU	1.70 (1.16, 2.49)	0.0062	PET DISCRETIZED SUVbwmin	1.58 (1.14, 2.19)	0.0056
			RP T1 CONVENTIONAL Skewness	1.38 (0.94, 2.02)	0.097
			RP T1 NGLDM Busyness	1.31 (1.01,1.70)	0.043

### Model performance

**Figure 3:**
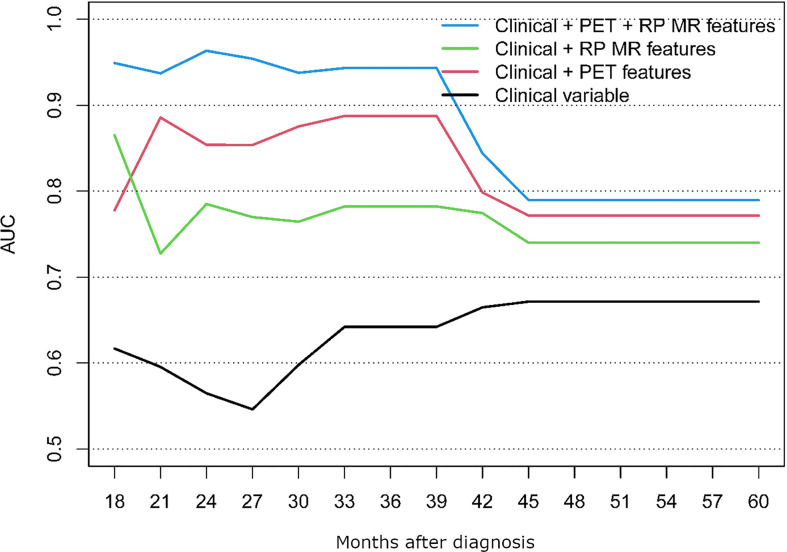
OS AUC comparison between the different prognostic models.

**Figure 4:**
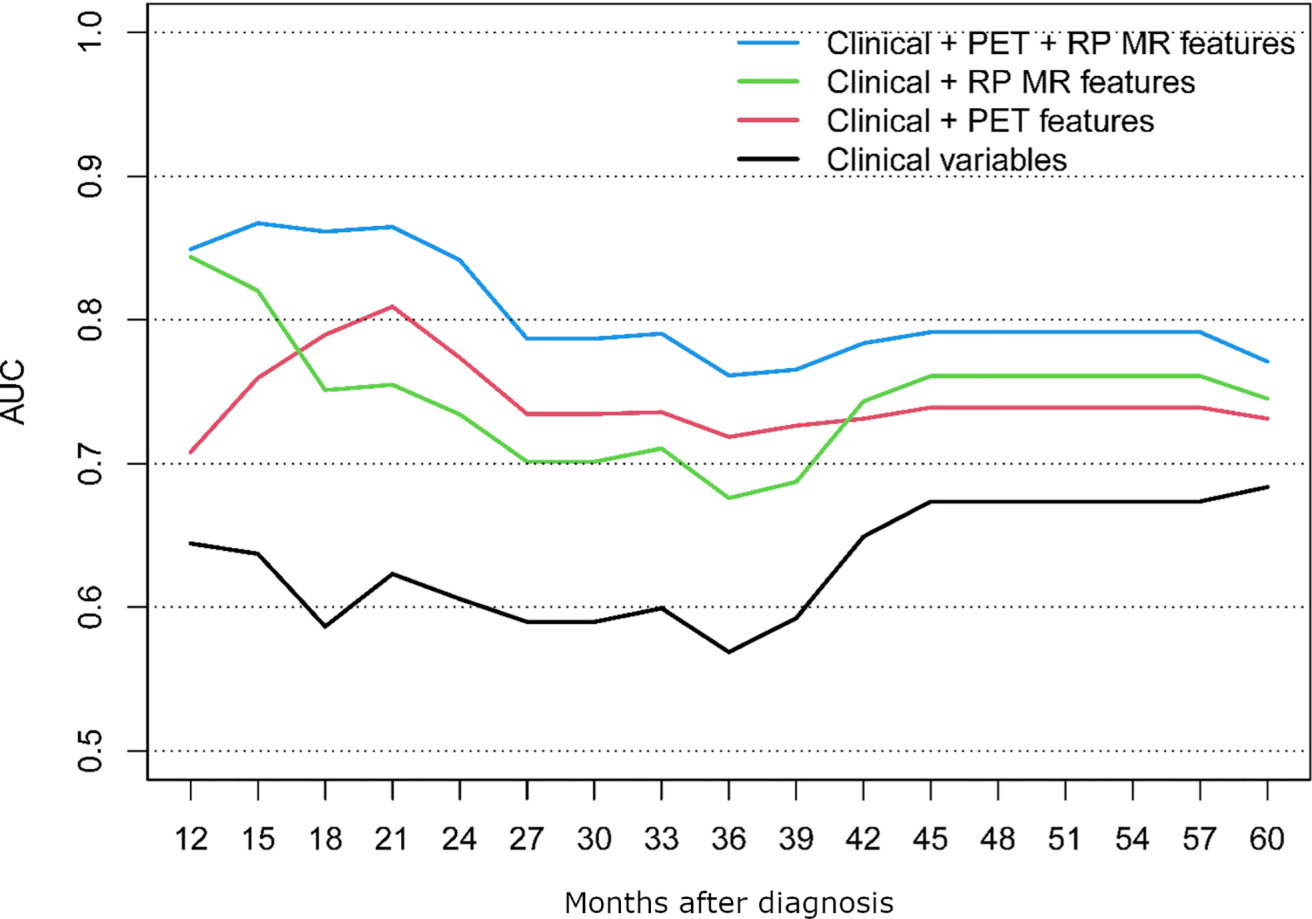
PFS AUC comparison between the different prognostic models.

The performance of the following models was compared; clinical alone, clinical + PET/CT features, clinical + RP-MR, and clinical + PET/CT + RP-MR, for both OS and PFS, as shown in **Figures 3**, **4**. In both situations, models considering clinical + PET/CT + RP-MR features outperformed those considering only clinical, clinical + PET/CT or clinical + RP-MR features (AUC 0.96 *vs* 0.56 *vs* 0.85 *vs* 0.79 at 24 months in OS and 0.86 *vs* 0.62 *vs* 0.81 *vs* 0.75 at 21 months in PFS), which suggests a synergy between PET/CT and RP-MR features. It is to be noted that in both the OS and PFS models, clinical + RP-MR features appear to initially outperform clinical + PET/CT features (AUC 0.87 *vs* 0.78 at 18 months in OS and AUC 0.82 *vs* 0.76 at 14 months in PFS). In the OS model, clinical + PET/CT outperformed clinical + RP-MR thereafter (AUC 0.89 *vs* 0.78 at 39 months), while in the PFS model, clinical + PET/CT features outperformed clinical + RP-MR features from 18 to 39 months (AUC 0.81 *vs* 0.75 at 21 months), with clinical + RP-MR outperforming those of clinical + PET/CT features from 42 months thereafter (AUC 0.76 *vs* 0.74 at 45 months).

## Discussion

To the best of our knowledge, no study so far has evaluated PET/CT combined with MR-based radiomics and baseline clinical parameters among patients with NPC. We identified that radiomic features from MR and PET/CT were associated with improved prediction of OS and PFS, particularly when combined (AUC of 0.96 and 0.86, respectively). Clinical + MR features initially outperformed those of Clinical + PET/CT (<18 months), with Clinical + PET/CT features then outperforming those of Clinical + RP-MR consistently in the OS model, while Clinical + RP-MR features subsequently outperformed those of Clinical + PET/CT (>42 months) in the PFS model.

Our study confirms the findings of multiple studies in the literature that have demonstrated the pre-treatment prognostic value of MR-based radiomics among patients with NPC, consistently showing that MR-based radiomics outperform clinical features alone when predicting either PFS or OS (4, 6, 11–20, 22). The AUC for clinical + RP-MR in our study was as high as 0.84 for PFS and 0.87 for OS, which is comparable with the literature where AUC varies from 0.8 (18) to 0.886 (12), and the C-index from 0.72 (19) to 0.874 (20).

A significant proportion of these studies were only performed among patients with advanced (stages III–IV), non-metastatic NPC (4, 6, 11–14), with the remainder performed among non-metastatic NPC patients of all stages, similar to our study (15, 17–20, 22).

Similar to the majority of MR-based radiomic studies, we included both contrast-enhanced T1-w and T2-w MR sequences in our study (4, 6, 11–14, 16–18, 20, 22). However, although both contrast-enhanced T1-w and T2-w MR sequences were evaluated, ultimately only radiomic features from the contrast enhanced T1-w sequences were found to be significant and included in our final OS and PFS models (RP_T1_GLZLM_GLNU, RP T1 CONVENTIONAL Skewness, and RP T1 NGLDM Busyness). This is partly different when compared to other studies which have shown that joint contrast-enhanced T1 and T2 radiomic features have a better prognostic performance than T1 or T2 features alone and may be as a result of better performing PET-based radiomic features being incorporated into our model (11, 12).

Another differentiation compared to the literature are the methods used for radiomic feature extraction (e.g., MATLAB), with only one other NPC radiomic study also using LIFEx software for radiomic feature extraction (14). Despite utilization of the same MR sequences (contrast enhanced T1-w and T2-w sequences) and radiomic extraction software, different radiomic features were found to be significant [RP_T1_GLZLM_GLNU, RP T1 CONVENTIONAL Skewness, and RP T1 NGLDM Busyness in our study, and GLCM_Energy, GLCM_Corre, and CONV_st in (14)]. This may reflect our utilization of 3.0 T fat-saturated MR sequences with different technical parameters. Similar to the majority of studies into NPC radiomics, our study evaluated radiomic parameters within the primary tumor. However, there are a number of studies that assess both the primary NPC tumor and adjacent locoregional lymph nodes, with similar findings, confirming the prognostic value of combined baseline clinical and MR-based radiomics (14, 19).

There are three studies in the literature exploring the performance of PET/CT based radiomic features among NPC patients. Similar to our study, they demonstrated that combined clinical with PET/CT features improved the prediction of PFS with a c-index of 0.77 (23), 0.69 (24), and an AUC of 0.829 (7) compared with 0.81 in our study. The study from Peng et al. only examined patients with advanced NPC (stages II–IV) (7), compared with ours and the remaining PET/CT radiomic studies. In the study by Lv et al. age was identified as a significant clinical parameter, as in our study, in addition to IgA, N, and M stage (23). Our study identified PET_CONVENTIONAL_SUVbwQ1 and PET DISCRETIZED SUVbwmin as significant PET radiomic parameters, but no PET features were retained following multivariable analysis in the study of Lv et al. (23). By comparison, other parameters like PET-NGTDM-Complexity, CT-GLGLM-LGGE, and PET-GLGLM-SGLGE were found to be significant in the study by Xu et al. (24).

Our study evaluated both the PET and the CT components of the PET/CT study, but no CT parameters were found to have significant prognostic value in our study, unlike the remaining PET/CT-based radiomic studies (7, 23, 24). We routinely evaluate the CT component in our radiomics studies since PET/CT is used clinically as a combined imaging modality. The complementary value of the CT component has previously been demonstrated in the literature (27), and if radiomics should ever make it into clinical routine decision-making in the future, then the combined value of PET and CT radiomics would be beneficial per disease site.

There is currently only a single study examining the prognostic value between PET and MR in the existing literature (5), however, this only utilizes T2-w MR and PET images. Our study is the first demonstrating the improved prognostic value of combined clinical + PET/CT + MR features compared with clinical, PET/CT, or MR features individually for both OS and PFS (AUC 0.96 at 24 months in OS and 0.86 at 21 months in PFS). Since our results indicated that mainly PET and MR radiomic features seem to have a prognostic value, combined PET/MR imaging could be considered as a clinical tool for staging, prognostication, and potentially surveillance of NPC. This may offer the patient (and the hospital) improved staging logistics (one combined exam compared to PET/CT and MR separately) as well as possibly a better prognostication tool in the future.

Interestingly, clinical + RP-MR features initially outperformed clinical + PET/CT for both OS and PFS in the follow up period (<18 months), and for PFS (>42 months). Since MRI is used mostly for local staging (because of its well-documented superiority), one consideration is that the local tumor may potentially be the dominant driver and dictate short-term tumoral behavior. PET, however, may provide improved overall prognostication, representing the overall pathophysiological behavior in a better way than morphological imaging procedures. Ultimately, these findings remain indeterminate and would need to be confirmed in similar studies.

Our study had some limitations, predominantly in terms of methodology. This was a retrospective study with a moderate number of patients (124) [sample sizes ranged from 85 to 737 subjects in the literature (3)], with mixed clinical stages of NPC (I–IV). Other prognostic molecular biomarkers, such as hemoglobin, LDH, neutrophil–lymphocyte ration, c-Met, ERBB3, and MTDH, were not available for inclusion in the study (21). These were not routinely obtained among our patient cohort, at our institution, at the time of treatment.

Although the PET/CT and RP-MR images were obtained from the same institution and scanners, maintaining uniformity in image acquisition, no image preprocessing was performed prior to segmentation. However, there is currently no general consensus available regarding whether and which image preprocessing should be performed. Some researchers are even opposed to image preprocessing since it would be prohibitive to implement clinically on a large scale. Also related to study acquisition, CT was performed without intravenous contrast, which could have contributed to its failure to produce significant radiomic features, although other studies in different cancer entities actually did find prognostic value in the CT component of PET/CT. Finally, segmentation was also only performed manually for CT and MR, without reproducibility evaluation.

Statistical methodology, in terms of feature selection and modeling, is highly variable between radiomic studies (LASSO, RFE, univariable analysis; RS, CR, and nomogram; Chi-squared test, SFFS, and SVM). We performed a univariable analysis followed by the construction of multivariable Cox regression models into which radiomic features were then added. This approach allowed us to identify prognostic factors by using interpretable models. A major difference between our studies and those in the literature is that the majority of studies use both training and validation cohorts to assess model performance, with only one other study utilizing internal cross-validation (19). Thus, the lack of an external validation cohort is a potential limitation of our study, and therefore, future/other studies would be needed to further validate our results. Because of the absence of an independent validation cohort, this study can only be classified as explorative (19).

## Conclusions

In conclusion, our study demonstrated that PET/CT-based radiomic features may improve survival prognostication when combined with baseline clinical and MR-based radiomic features among NPC patients.

## Data availability statement

The raw data supporting the conclusions of this article will be made available by the authors, without undue reservation.

## Ethics statement

This study was reviewed and approved by the UHN Ethics Committee. Written informed consent for participation was not required for this study in accordance with the national legislation and the institutional requirements.

## Author contributions

All authors contributed to the article and approved the submitted version.

## Conflict of interest

The authors declare that the research was conducted in the absence of any commercial or financial relationships that could be construed as a potential conflict of interest.

## Publisher’s note

All claims expressed in this article are solely those of the authors and do not necessarily represent those of their affiliated organizations, or those of the publisher, the editors and the reviewers. Any product that may be evaluated in this article, or claim that may be made by its manufacturer, is not guaranteed or endorsed by the publisher.
