# Supplementary material for: Prognostic value of PET/CT and MR-based baseline radiomics among patients with non-metastatic nasopharyngeal carcinoma
Source: Front Oncol. 2022 Oct 24;12:952763. doi: 10.3389/fonc.2022.952763 (PMC9638017; doi:10.3389/fonc.2022.952763)
Supplement: Supplementary file 1 [file Table_1.docx]

**SUPPLEMENTARY TABLES**

Table S1. Radiomic imaging features extracted by sequence-type.

|  |  | **Radiotherapy Planning MRI (RP-MRI)** | |
| --- | --- | --- | --- |
| **PET** | **CT** | **Contrast enhanced fat saturated T1** | **Contrast enhanced fat saturated T2** |
| CONVENTIONAL_SUVbwmin | CONVENTIONAL_HUmin | CONVENTIONAL_min | CONVENTIONAL_min |
| CONVENTIONAL_SUVbwmean | CONVENTIONAL_HUmean | CONVENTIONAL_mean | CONVENTIONAL_mean |
| CONVENTIONAL_SUVbwstd | CONVENTIONAL_HUstd | CONVENTIONAL_std | CONVENTIONAL_std |
| CONVENTIONAL_SUVbwmax | CONVENTIONAL_HUmax | CONVENTIONAL_max | CONVENTIONAL_max |
| CONVENTIONAL_SUVbwQ1 | CONVENTIONAL_HUQ1 | CONVENTIONAL_Q1 | CONVENTIONAL_Q1 |
| CONVENTIONAL_SUVbwQ2 | CONVENTIONAL_HUQ2 | CONVENTIONAL_Q2 | CONVENTIONAL_Q2 |
| CONVENTIONAL_SUVbwQ3 | CONVENTIONAL_HUQ3 | CONVENTIONAL_Q3 | CONVENTIONAL_Q3 |
| CONVENTIONAL_SUVbwSkewness | CONVENTIONAL_HUSkewness | CONVENTIONAL_Skewness | CONVENTIONAL_Skewness |
| CONVENTIONAL_SUVbwKurtosis | CONVENTIONAL_HUKurtosis | CONVENTIONAL_Kurtosis | CONVENTIONAL_Kurtosis |
| CONVENTIONAL_SUVbwExcessKurtosis | CONVENTIONAL_HUExcessKurtosis | CONVENTIONAL_ExcessKurtosis | CONVENTIONAL_ExcessKurtosis |
| CONVENTIONAL_SUVbwpeakSphere0.5mL:discretized volume sought | CONVENTIONAL_HUpeakSphere0.5mL:discretized volume sought | CONVENTIONAL_peakSphere0.5mL:discretized volume sought | CONVENTIONAL_peakSphere0.5mL:discretized volume sought |
| CONVENTIONAL_SUVbwpeakSphere0.5mL(value only for PET or NM) | CONVENTIONAL_HUpeakSphere0.5mL(value only for PET or NM) | CONVENTIONAL_peakSphere0.5mL(value only for PET or NM) | CONVENTIONAL_peakSphere0.5mL(value only for PET or NM) |
| CONVENTIONAL_SUVbwpeakSphere1mL:discretized volume sought | CONVENTIONAL_HUpeakSphere1mL:discretized volume sought | CONVENTIONAL_peakSphere1mL:discretized volume sought | CONVENTIONAL_peakSphere1mL:discretized volume sought |
| CONVENTIONAL_SUVbwpeakSphere1mL(value only for PET or NM) | CONVENTIONAL_HUpeakSphere1mL(value only for PET or NM) | CONVENTIONAL_peakSphere1mL(value only for PET or NM) | CONVENTIONAL_peakSphere1mL(value only for PET or NM) |
| CONVENTIONAL_SUVbwcalciumAgatstonScore[onlyForCT] | CONVENTIONAL_HUcalciumAgatstonScore[onlyForCT] | CONVENTIONAL_calciumAgatstonScore[onlyForCT] | CONVENTIONAL_calciumAgatstonScore[onlyForCT] |
| CONVENTIONAL_TLG(mL)[onlyForPETorNM] | CONVENTIONAL_TLG(mL)[onlyForPETorNM] | CONVENTIONAL_TLG(mL)[onlyForPETorNM] | CONVENTIONAL_TLG(mL)[onlyForPETorNM] |
| CONVENTIONAL_RIM_SUVbwmin | CONVENTIONAL_RIM_HUmin | CONVENTIONAL_RIM_min | CONVENTIONAL_RIM_min |
| CONVENTIONAL_RIM_SUVbwmean | CONVENTIONAL_RIM_HUmean | CONVENTIONAL_RIM_mean | CONVENTIONAL_RIM_mean |
| CONVENTIONAL_RIM_SUVbwstdev | CONVENTIONAL_RIM_HUstdev | CONVENTIONAL_RIM_stdev | CONVENTIONAL_RIM_stdev |
| CONVENTIONAL_RIM_SUVbwmax | CONVENTIONAL_RIM_HUmax | CONVENTIONAL_RIM_max | CONVENTIONAL_RIM_max |
| CONVENTIONAL_RIM_SUVbwVolume(mL) | CONVENTIONAL_RIM_HUVolume(mL) | CONVENTIONAL_RIM_Volume(mL) | CONVENTIONAL_RIM_Volume(mL) |
| CONVENTIONAL_RIM_SUVbwVolume(vx) | CONVENTIONAL_RIM_HUVolume(vx) | CONVENTIONAL_RIM_Volume(vx) | CONVENTIONAL_RIM_Volume(vx) |
| CONVENTIONAL_RIM_SUVbwsum | CONVENTIONAL_RIM_HUsum | CONVENTIONAL_RIM_sum | CONVENTIONAL_RIM_sum |
| DISCRETIZED_SUVbwmin | DISCRETIZED_HUmin | DISCRETIZED_min | DISCRETIZED_min |
| DISCRETIZED_SUVbwmean | DISCRETIZED_HUmean | DISCRETIZED_mean | DISCRETIZED_mean |
| DISCRETIZED_SUVbwstd | DISCRETIZED_HUstd | DISCRETIZED_std | DISCRETIZED_std |
| DISCRETIZED_SUVbwmax | DISCRETIZED_HUmax | DISCRETIZED_max | DISCRETIZED_max |
| DISCRETIZED_SUVbwQ1 | DISCRETIZED_HUQ1 | DISCRETIZED_Q1 | DISCRETIZED_Q1 |
| DISCRETIZED_SUVbwQ2 | DISCRETIZED_HUQ2 | DISCRETIZED_Q2 | DISCRETIZED_Q2 |
| DISCRETIZED_SUVbwQ3 | DISCRETIZED_HUQ3 | DISCRETIZED_Q3 | DISCRETIZED_Q3 |
| DISCRETIZED_SUVbwSkewness | DISCRETIZED_HUSkewness | DISCRETIZED_Skewness | DISCRETIZED_Skewness |
| DISCRETIZED_SUVbwKurtosis | DISCRETIZED_HUKurtosis | DISCRETIZED_Kurtosis | DISCRETIZED_Kurtosis |
| DISCRETIZED_SUVbwExcessKurtosis | DISCRETIZED_HUExcessKurtosis | DISCRETIZED_ExcessKurtosis | DISCRETIZED_ExcessKurtosis |
| DISCRETIZED_SUVbwpeakSphere0.5mL:discretized volume sought | DISCRETIZED_HUpeakSphere0.5mL:discretized volume sought | DISCRETIZED_peakSphere0.5mL:discretized volume sought | DISCRETIZED_peakSphere0.5mL:discretized volume sought |
| DISCRETIZED_SUVbwpeakSphere0.5mL(value only for PET or NM) | DISCRETIZED_HUpeakSphere0.5mL(value only for PET or NM) | DISCRETIZED_peakSphere0.5mL(value only for PET or NM) | DISCRETIZED_peakSphere0.5mL(value only for PET or NM) |
| DISCRETIZED_SUVbwpeakSphere1mL:discretized volume sought | DISCRETIZED_HUpeakSphere1mL:discretized volume sought | DISCRETIZED_peakSphere1mL:discretized volume sought | DISCRETIZED_peakSphere1mL:discretized volume sought |
| DISCRETIZED_SUVbwpeakSphere1mL(value only for PET or NM) | DISCRETIZED_HUpeakSphere1mL(value only for PET or NM) | DISCRETIZED_peakSphere1mL(value only for PET or NM) | DISCRETIZED_peakSphere1mL(value only for PET or NM) |
| DISCRETIZED_TLG(mL)[onlyForPETorNM] | DISCRETIZED_TLG(mL)[onlyForPETorNM] | DISCRETIZED_TLG(mL)[onlyForPETorNM] | DISCRETIZED_TLG(mL)[onlyForPETorNM] |
| DISCRETIZED_HISTO_Entropy_log10 | DISCRETIZED_HISTO_Entropy_log10 | DISCRETIZED_HISTO_Entropy_log10 | DISCRETIZED_HISTO_Entropy_log10 |
| DISCRETIZED_HISTO_Entropy_log2 | DISCRETIZED_HISTO_Entropy_log2 | DISCRETIZED_HISTO_Entropy_log2 | DISCRETIZED_HISTO_Entropy_log2 |
| DISCRETIZED_HISTO_Energy[=Uniformity] | DISCRETIZED_HISTO_Energy[=Uniformity] | DISCRETIZED_HISTO_Energy[=Uniformity] | DISCRETIZED_HISTO_Energy[=Uniformity] |
| DISCRETIZED_RIM_SUVbwmin | DISCRETIZED_RIM_HUmin | DISCRETIZED_RIM_min | DISCRETIZED_RIM_min |
| DISCRETIZED_RIM_SUVbwmean | DISCRETIZED_RIM_HUmean | DISCRETIZED_RIM_mean | DISCRETIZED_RIM_mean |
| DISCRETIZED_RIM_SUVbwstdev | DISCRETIZED_RIM_HUstdev | DISCRETIZED_RIM_stdev | DISCRETIZED_RIM_stdev |
| DISCRETIZED_RIM_SUVbwmax | DISCRETIZED_RIM_HUmax | DISCRETIZED_RIM_max | DISCRETIZED_RIM_max |
| DISCRETIZED_RIM_SUVbwsum | DISCRETIZED_RIM_HUsum | DISCRETIZED_RIM_sum | DISCRETIZED_RIM_sum |
| SHAPE_Volume(mL) | SHAPE_Volume(mL) | SHAPE_Volume(mL) | SHAPE_Volume(mL) |
| SHAPE_Volume(vx) | SHAPE_Volume(vx) | SHAPE_Volume(vx) | SHAPE_Volume(vx) |
| SHAPE_Sphericity[onlyFor3DROI]) | SHAPE_Sphericity[onlyFor3DROI]) | SHAPE_Sphericity[onlyFor3DROI]) | SHAPE_Sphericity[onlyFor3DROI]) |
| SHAPE_Surface(mm2)[onlyFor3DROI] | SHAPE_Surface(mm2)[onlyFor3DROI] | SHAPE_Surface(mm2)[onlyFor3DROI] | SHAPE_Surface(mm2)[onlyFor3DROI] |
| SHAPE_Compacity[onlyFor3DROI] | SHAPE_Compacity[onlyFor3DROI] | SHAPE_Compacity[onlyFor3DROI] | SHAPE_Compacity[onlyFor3DROI] |
| PARAMS_DistanceOfNeighbours | PARAMS_DistanceOfNeighbours | PARAMS_DistanceOfNeighbours | PARAMS_DistanceOfNeighbours |
| PARAMS_NumberOfGreyLevels | PARAMS_NumberOfGreyLevels | PARAMS_NumberOfGreyLevels | PARAMS_NumberOfGreyLevels |
| PARAMS_BinSize | PARAMS_BinSize | PARAMS_BinSize | PARAMS_BinSize |
| PARAMS_IntensityResampling | PARAMS_IntensityResampling | PARAMS_IntensityResampling | PARAMS_IntensityResampling |
| PARAMS_BoundsRangeOfValueAfterDiscretisation(SUVbw) | PARAMS_BoundsRangeOfValueAfterDiscretisation(HU) | PARAMS_BoundsRangeOfValueAfterDiscretisation() | PARAMS_BoundsRangeOfValueAfterDiscretisation() |
| PARAMS_ZSpatialResampling | PARAMS_ZSpatialResampling | PARAMS_ZSpatialResampling | PARAMS_ZSpatialResampling |
| PARAMS_YSpatialResampling | PARAMS_YSpatialResampling | PARAMS_YSpatialResampling | PARAMS_YSpatialResampling |
| PARAMS_XSpatialResampling | PARAMS_XSpatialResampling | PARAMS_XSpatialResampling | PARAMS_XSpatialResampling |
| CHECK_Cluster(s)ToSmall | CHECK_Cluster(s)ToSmall | CHECK_Cluster(s)ToSmall | CHECK_Cluster(s)ToSmall |
| GLCM_Homogeneity[=InverseDifference] | GLCM_Homogeneity[=InverseDifference] | GLCM_Homogeneity[=InverseDifference] | GLCM_Homogeneity[=InverseDifference] |
| GLCM_Energy[=AngularSecondMoment] | GLCM_Energy[=AngularSecondMoment] | GLCM_Energy[=AngularSecondMoment] | GLCM_Energy[=AngularSecondMoment] |
| GLCM_Contrast[=Variance] | GLCM_Contrast[=Variance] | GLCM_Contrast[=Variance] | GLCM_Contrast[=Variance] |
| GLCM_Correlation | GLCM_Correlation | GLCM_Correlation | GLCM_Correlation |
| GLCM_Entropy_log10 | GLCM_Entropy_log10 | GLCM_Entropy_log10 | GLCM_Entropy_log10 |
| GLCM_Entropy_log2[=JointEntropy] | GLCM_Entropy_log2[=JointEntropy] | GLCM_Entropy_log2[=JointEntropy] | GLCM_Entropy_log2[=JointEntropy] |
| GLCM_Dissimilarity | GLCM_Dissimilarity | GLCM_Dissimilarity | GLCM_Dissimilarity |
| GLRLM_SRE | GLRLM_SRE | GLRLM_SRE | GLRLM_SRE |
| GLRLM_LRE | GLRLM_LRE | GLRLM_LRE | GLRLM_LRE |
| GLRLM_LGRE | GLRLM_LGRE | GLRLM_LGRE | GLRLM_LGRE |
| GLRLM_HGRE | GLRLM_HGRE | GLRLM_HGRE | GLRLM_HGRE |
| GLRLM_SRLGE | GLRLM_SRLGE | GLRLM_SRLGE | GLRLM_SRLGE |
| GLRLM_SRHGE | GLRLM_SRHGE | GLRLM_SRHGE | GLRLM_SRHGE |
| GLRLM_LRLGE | GLRLM_LRLGE | GLRLM_LRLGE | GLRLM_LRLGE |
| GLRLM_LRHGE | GLRLM_LRHGE | GLRLM_LRHGE | GLRLM_LRHGE |
| GLRLM_GLNU | GLRLM_GLNU | GLRLM_GLNU | GLRLM_GLNU |
| GLRLM_RLNU | GLRLM_RLNU | GLRLM_RLNU | GLRLM_RLNU |
| GLRLM_RP | GLRLM_RP | GLRLM_RP | GLRLM_RP |
| NGLDM_Coarseness | NGLDM_Coarseness | NGLDM_Coarseness | NGLDM_Coarseness |
| NGLDM_Contrast | NGLDM_Contrast | NGLDM_Contrast | NGLDM_Contrast |
| NGLDM_Busyness | NGLDM_Busyness | NGLDM_Busyness | NGLDM_Busyness |
| GLZLM_SZE | GLZLM_SZE | GLZLM_SZE | GLZLM_SZE |
| GLZLM_LZE | GLZLM_LZE | GLZLM_LZE | GLZLM_LZE |
| GLZLM_LGZE | GLZLM_LGZE | GLZLM_LGZE | GLZLM_LGZE |
| GLZLM_HGZE | GLZLM_HGZE | GLZLM_HGZE | GLZLM_HGZE |
| GLZLM_SZLGE | GLZLM_SZLGE | GLZLM_SZLGE | GLZLM_SZLGE |
| GLZLM_SZHGE | GLZLM_SZHGE | GLZLM_SZHGE | GLZLM_SZHGE |
| GLZLM_LZLGE | GLZLM_LZLGE | GLZLM_LZLGE | GLZLM_LZLGE |
| GLZLM_LZHGE | GLZLM_LZHGE | GLZLM_LZHGE | GLZLM_LZHGE |
| GLZLM_GLNU | GLZLM_GLNU | GLZLM_GLNU | GLZLM_GLNU |
| GLZLM_ZLNU | GLZLM_ZLNU | GLZLM_ZLNU | GLZLM_ZLNU |
| GLZLM_ZP | GLZLM_ZP | GLZLM_ZP | GLZLM_ZP |
| TimePosition | TimePosition | TimePosition | TimePosition |
| zLocation[onlyFor2DROI] | zLocation[onlyFor2DROI] | zLocation[onlyFor2DROI] | zLocation[onlyFor2DROI] |
